# Are the relationships between mental health issues and being left-behind gendered in China: A systematic review and meta-analysis

**DOI:** 10.1371/journal.pone.0279278

**Published:** 2023-04-13

**Authors:** Jason Hung, Jackson Chen, Olivia Chen

**Affiliations:** 1 Department of Sociology, The University of Cambridge, Cambridge, Cambridgeshire, United Kingdom; 2 Institute of Sociology, Academia Sinica, Taipei City, Taiwan; 3 Department of Sociology, The London School of Economics, London, Greater London, United Kingdom; 4 Department of Social Policy, The London School of Economics, London, Greater London, United Kingdom; Chiang Mai University, THAILAND

## Abstract

**Background:**

While most existing studies reveal left-behind children (LBC) are prone to suffering from mental health issues, some other literature fails to develop a statistical significance between being left-behind and facing mental health dilemmas. In further detail, it is noteworthy that suicide ideation is a gendered issue. Here girls, relative to their male counterparts, are more likely to experience emotional and affective challenges, alongside a higher risk of suicide ideation. Aside from suicide ideation, the rate of suicide attempts is also higher among Chinese female than among male LBC. However, Chang et al. counter-argue that, within the LBC cohorts, it is not statistically significant to state that girls were more likely for suicide attempts than boys.

**Methods:**

In this paper, a systematic review of relevant literature and a meta-analysis of all qualified randomised controlled trial (RCT) studies were conducted. The authors aim to examine all relevant studies with similar methodologies to observe the nuanced relationships between being left-behind and mental health issues in Chinese contexts. Specifically, the authors will, grounded on the findings from the systematic review and meta-analysis, assess whether the relationship between mental health issues and being left-behind is gendered in Chinese contexts by analysing all relevant findings derived from similar methodologies and the same method (i.e., RCT).

**Results:**

Aside from Wanjie et al.’s studies, it is noticeable that the rest of the studies share similar point estimates and their CIs overlapped to a large extent. As per the I^2^, given the presence of Wanjie et al.’s studies that demonstrate an observably higher degree of heterogeneity than the rest of the studies, the I^2^ values, each for the measurement of anxiety and depression, are 74.8 percent and 34.7 percent respectively. This shows that there is a considerable heterogeneity level for anxiety, while the heterogeneity level for depression is moderate. However, both p-values for the I^2^ statistics are larger than 0.05. Therefore, at the 0.05 significance level, it is statistically insignificant to reject the null hypothesis that there is no heterogeneity between individual studies in both the subgroups of anxiety and depression. Therefore, the concern of the potentially substantial heterogeneity should be irrelevant in this meta-analysis.

Beyond the discussion from the forest plot, when looking at the single study addressing the relationship between being left-behind and having suicide attempts (note: LBC—OR is 1.22; 95 percent CI is 1.22 –and NLBC—OR is 1.42; 95 percent CI is 1.09–1.86 –at the p-value of 0.34), the findings demonstrate that such a relationship *per se* is not gendered at the 0.05 statistical significance level.

However, when examining the relationship between being resilient and left-behind, such an association is gendered where the OR of female left-behind university students being resilient, relative to male left-behind university students, is slightly higher than that of female non-left-behind university students being resilient, relative to their male non-left-behind university student counterparts. It is noteworthy that this study focuses on studying left-behind and non-left-behind samples who entered universities. Since a raft of LBC are socially, educationally disadvantaged, they lack the opportunities to receive higher education. Therefore, the findings of this study might not be indicative of the LBC population at large.

**Conclusions:**

While the findings of this meta-analysis project fail to reflect any gendered issues statistically, the authors are aware of the fact that the data included in this project were collected based on perception. Here samples, or their parents and teachers, were responsible for answering the questions with respect to samples’ mental health status and demographic details. In China, especially in less developed rural regions, the discourse on mental health challenges might continue to be seen as taboo, so individuals giving responses might, consciously or not, tend to give socially desirable answers to avoid any potential social stigmatisation. Therefore, there is some extent of reservation regarding the validity of the included studies’ data.

## Introduction

Per the United Nations Children’s Fund, millions of children are left-behind by one parent or both parents who migrate to find work and leave their children behind in home countries or countries of habitual residence. Being left-behind has caused entrenched detrimental impacts on children’s development, opportunities, economic status and wellbeing [1]. In 2015, in China specifically, over 68 million children, comprising 25 percent of the child population nationwide, were left behind by at least one of their parents. Some 70 percent of LBC failed to see their migrated parents for the entire year. Additionally, those who were able to see their migrated parents were usually subject to a very brief reunion during annual national holidays [2]. LBC is coined by Shangguan [3], initially and briefly referring to children who were taken care of by their grandparents when their parents relocated for work [4]. Since then, the understanding of LBC has been consolidated, addressing children under the age of 18 who reside at home while either or both of their parents migrate elsewhere over a period of at least six consecutive months [5].

Compared to non-left-behind children (NLBC), existing literature argues that LBC had 52, 70 and 85 percent higher risks of developing depression, suicide ideation and anxiety respectively [6]. Additional literature presents that LBC display 34 and nine percent higher risks of suicide ideation and attempts respectively. These circumstances are primarily attributed to the lack of parental love and care throughout childhood and adolescence [4]. Supported by the Attachment Theory, LBC are prone to develop insecure attachment relationships imposed by long-term parental separation, because they often suffer from an absence or lack of love, lack of confidence, lack of security, exhaustion, confusion and anxiety [7]. Caused by long-term parent-child separation, LBC are more likely to present self-focused or self-oriented characteristics, serving as factors of depression and even suicide ideation [8, 9]. LBC are inclined to use death-related words, relative to their NLBC counterparts, on social media, symbolising the negativity that they possess [8].

## Mental health challenges and suicide among LBC

The development of depression is, in part, attributed to the experience of negative life events in the past. Compared to NLBC, LBC are more likely to suffer from depressive symptoms after their entry into adolescence [10]. Existing literature argues that LBC, with one or both of their parents migrated, experience 13.1 and 16.1 percent higher rates of depression respectively [7]. Here depression is viewed as the leading psychological risk factor of suicide among adolescence. To protect adolescents from depression, a higher level of entitlement to connection with perceived parents and families is a proven solution [11]. However, due to long-term parent-child separation, it is highly unlikely that LBC can enjoy sufficient connections with their family members, especially migrated parents.

Aside from depression, LBC have the proclivity to experience a range of adverse mental health symptoms. These include the feeling of insecurity, loneliness, anger, self-humiliation, low self-esteem, helplessness and low life satisfaction. Encountering any of these symptoms poses a higher risk of social withdrawal and suicide attempts among LBC throughout their childhood and adolescence [12]. With the absence of parenting, LBC lack role models they look up to or for supervision/guidance, promoting a higher degree of loneliness, sadness, hopelessness and alternative emotional distress [4, 2, 13]. As an additional note, in response to parental migration, teachers can be seen as the replaced role models for LBC, given existing literature’s argument that LBC usually hold positive attitudes towards their teachers due to the latter’s delivery of intellectual and affective guidance [14].

Moreover, some one-third of LBC in China suffer from panic symptoms. Without the disclosure of further detailed data, if panic symptoms are not addressed in a timely fashion, LBCs’ suffering may compound and general and social anxiety may be diagnosed [15]. Here LBC’s development of panic symptoms is partially caused by parental absence and long-term personal and academic difficulties—situations that prompt their prolonged, continual encounter of fear, then anxiety and depression, and ultimately even suicide [15]. Lu et al. [16] support such a discourse by conducting a RCT to find out whether suicide ideation was positively correlated to social anxiety and negatively correlated to self-esteem.

### Suicidality

The model of suicidality is widely considered as containing four stages in sequence, suicide ideation, suicide plan, suicide attempt and completed suicide [11]. Li et al. [13] argue that the absence of only the father or both parents raised the LBC’s risks of suffering from psychiatric morbidity and diagnosis of anxiety and depression; while the absence of only the mother enhanced their risks of suicide attempts or self-harm. Liu et al. [14] echo by stating that LBC exhibit up to 57.4 percent higher rate of psychological behavioural issues, including suicide ideation, relative to their NLBC counterparts.

Among LBC in China, some 34 percent have suicide tendencies, in addition to nine percent who committed suicide [4]. Yang et al. [17] further claim that being left behind by parent(s) showed no positive benefits to LBC’s mental health in their assessment. An additional study conducted by Wang et al. [18] demonstrates that LBC not attending boarding schools experienced more suicidal thoughts and attempts and anxiety, relative to their counterparts residing on boarding campuses. This shows that having role models for guidance, care and communications is pivotal when optimising LBC’s mental health.

### Resilience

In response to LBC’s proclivity to suffering from mental health issues and exposure to suicide risks, resilience—consisting of social abilities such as empathy, communication, cooperation, self-efficacy, problem solving, goals and aspirations, and self-awareness—is known as a protective factor against suicide risks for those who experience trauma during childhood and adolescence [9]. Xiao et al. [11] support such a claim by arguing that resilience is a proven protective factor of suicide ideation. Their findings show that a higher level of resilience was associated with a 32 percent drop in lifetime suicide ideation and a 49 percent decline in one-week suicide ideation.

“Resilience positively affects life satisfaction and psychological distress through the mediation effects of self-esteem” [19]. Self-esteem plays a fatal role in LBC’s mental and physical development [16]. Having higher self-esteem and self-belief can moderate the effect of any perceived discrimination LBC face, and the relationships between stressful life events and (1) depression and (2) non-suicidal self-injury [20].

## Research questions

Zhou [21] summarised relevant studies and found that while most studies reveal LBC are prone to suffering from mental health issues, some other literature fails to develop a statistical significance between being left-behind and facing mental health dilemmas. In this paper, a systematic review of relevant literature and a meta-analysis of all qualified—will be discussed below—RCT studies were conducted. The authors aim to examine all relevant studies with similar methodologies to observe the nuanced relationships between being left-behind and mental health issues in Chinese contexts.

In further detail, it is noteworthy that suicide ideation is a gendered issue. Here girls, relative to their male counterparts, are more likely to experience emotional and affective challenges, alongside a higher risk of suicide ideation [11, 22, 23]. Aside from suicide ideation, the rate of suicide attempts is also higher among Chinese female than among male LBC [10]. However, Chang et al. [24] counter-argue that, within the LBC cohorts, it is not statistically significant to state that girls were more likely for suicide attempts than boys. Specifically, the authors will, grounded on the findings from the systematic review and meta-analysis, assess whether the relationship between mental health issues and being left-behind is gendered in Chinese contexts by analysing all relevant findings derived from similar methodologies and the same method (i.e., RCT).

In the discussion and conclusions sections, the authors will further address the shortcomings of this meta-analysis by failing to examine any pooled studies on the risk of suicide and resilience. Yet, how the findings from non-pooled studies imply the scholarly and statistical discourse on the risk of suicide and resilience will be assessed.

## Materials and methods

**Fig 1:**
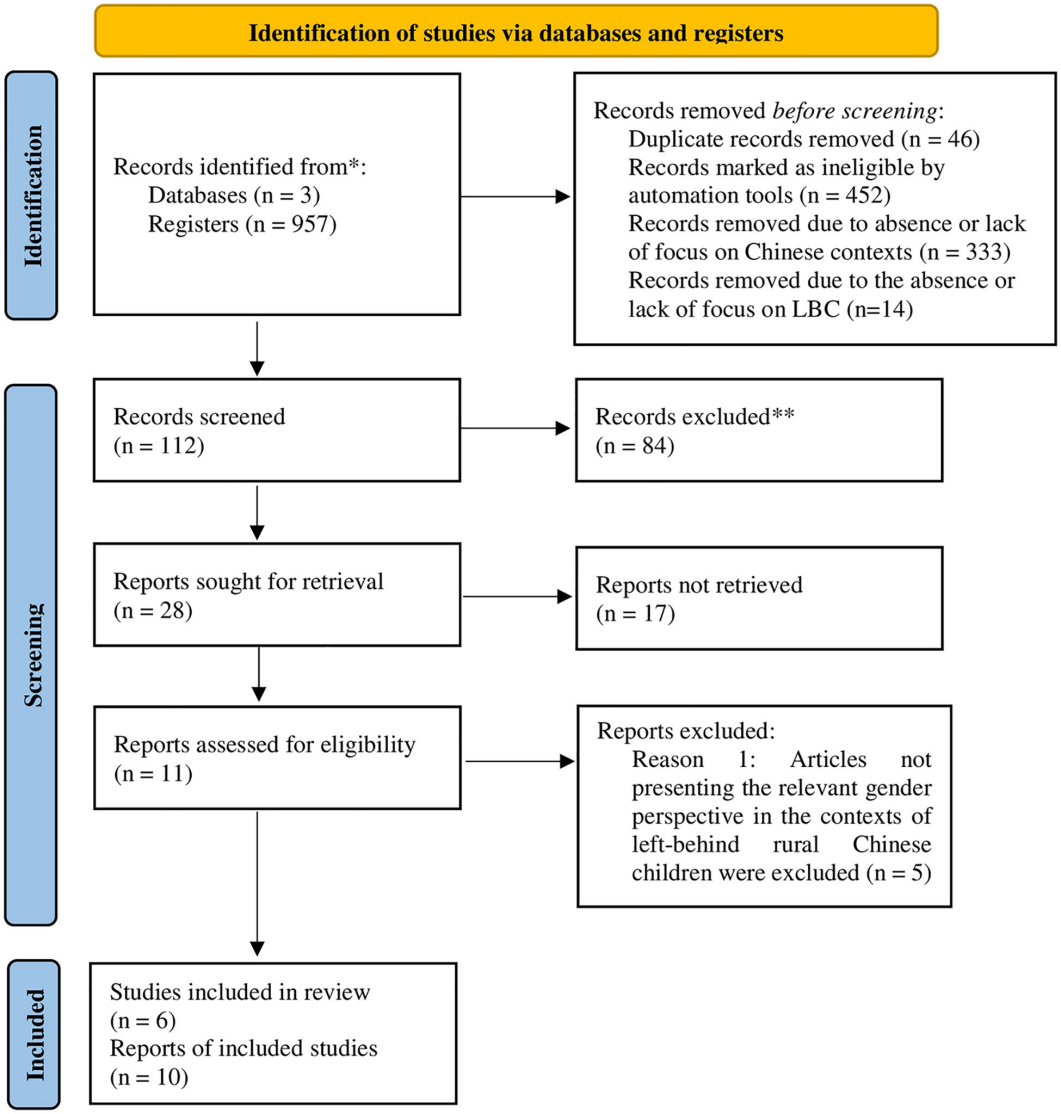
PRISMA 2020 flow diagram for new systematic reviews which included searches of databases and registers only. *Consider, if feasible to do so, reporting the number of records identified from each database or register searched (rather than the total number across all databases/registers). **If automation tools were used, indicate how many records were excluded by a human and how many were excluded by automation tools.

The meta-analysis followed the Preferred Reporting Items for Systematic Reviews and Meta-Analyses guidelines (see Fig 1).

### Literature search strategy

A computerised literature search was exercised to identify studies that were relevant to the research questions via the use of English bibliographical databases Google Scholar, Web of Science and Scopus. Studies published on or before 25^th^ June 2022 were screened and examined, if applicable. The following search terms were entered into the search engines: (“suicide” OR “suicidal”) & (“mental health” OR “psychological wellbeing”) & (“left-behind children” OR “leftover children” OR “LBC”) & (“China” OR “Chinese”). The search was restricted to English language publications only. All literature in Chinese or alternative, non-English languages were excluded. The bibliographies of the searched studies underwent a multi-stage screening to identify the most relevant, methodologically standardised research papers for meta-analysis.

### Eligibility criteria

The authors included (subsets of the) studies addressing the discourse on suicide and mental health challenges faced by LBC in Chinese contexts. Titles and abstracts of the acquired studies from the used bibliographic databases were screened for potential eligibility. Then, the full texts of possibly eligible studies were reviewed to determine if they were included in this meta-analysis.

J.H. invited J.C. and O.C. to be involved in the paper screening process. All accessible displayed results from those three databases were downloaded and reorganised by J.H.. J.H. stored the records of the collected downloaded papers in a Microsoft Excel file. He shared the Microsoft Excel file to J.C. and O.C., where J.H., J.C. and O.C. screened the file independently. All three authors individually and collectively screened the titles and abstracts of publications identified by their database search to eliminate papers failing to satisfy the inclusion criteria. Discrepancies were resolved through consensus between the three authors. They acquired the full texts of studies passing their initial title and abstract screening. Among the studies obtained and reviewed, for inter-rater reliability, the reviewers observed some 92 percent agreement. Miles and Huberman (1994) recommend that at least 80 percent agreement should be established in order to achieve sufficient reliability [25]. The 92 percent rating displayed in this review process, therefore, should be deemed satisfactory.

A total of 14 downloaded papers contained some contexts about gendered mental health discussions. However, these contexts refer to Chinese children as a whole rather than Chinese LBC. After discussing among them, all three agreed to exclude those 14 papers in this systematic review. To conclude, all three decided only six publications were included in this systematic review.

**Fig 2:**
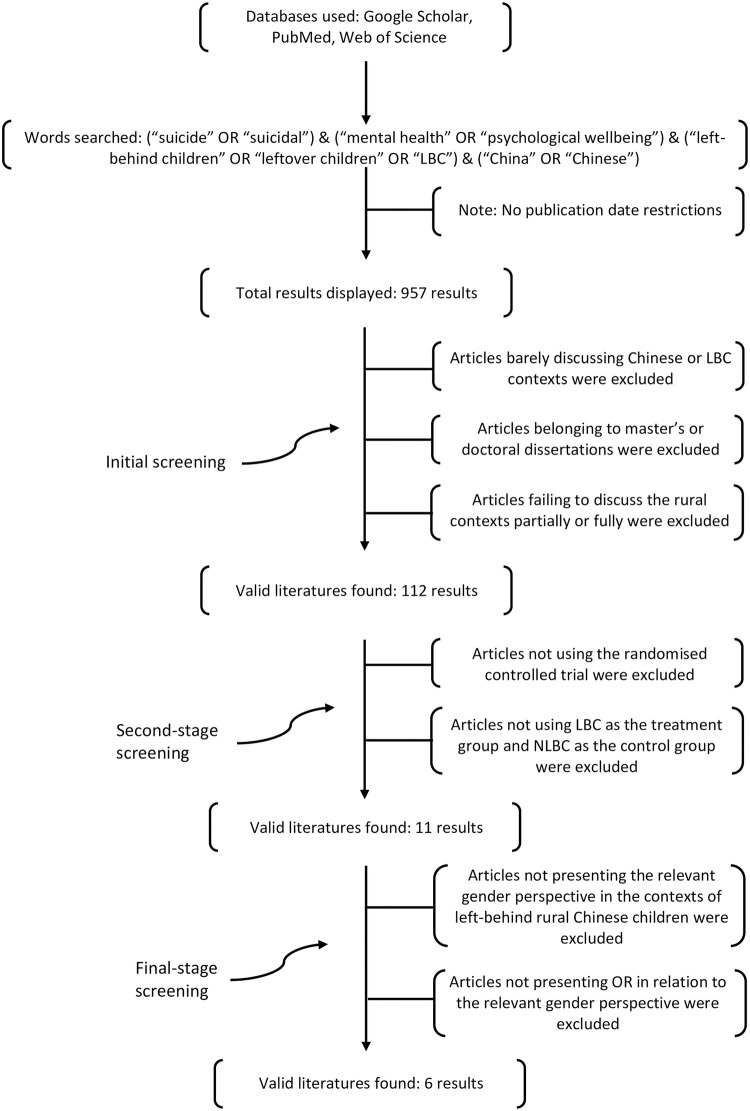
The systematic review process.

At the initial screening stage, articles barely discussing Chinese contexts, belonging to master’s or doctoral dissertations, and failing to discuss the rural contexts partially or in full were all excluded (see Fig 2). Here only studies addressing the encounters of risk of suicide and mental health challenges faced by LBC in rural, or both rural and urban, Chinese contexts were kept. At the second screening stage, articles not using the RCT, and not categorising LBC as the treatment group and NLBC as the control group were excluded. At the final-screening stage, articles not presenting the relevant gender perspective in the contexts of rural Chinese LBC, and not presenting the odds ratios (OR), or β coefficients, in relation to the relevant gender perspective, were excluded (See Fig 2). While studies conducting meta-analyses to address risks of suicide and mental health issues (such as depression and anxiety) by reviewing research projects that adopted a RCT already exist, to date, there is an absence of any meta-analyses that review existing studies exploiting a RCT to understand the gender perspective of risks of suicide and mental health issues in rural Chinese contexts. As a result, this meta-analysis project was developed.

### Data extraction and quality assessment

Data from included studies were extracted using a standardised form. First, in this paper, the following study traits were noted: name(s) of the author(s), year(s) of data collection, province of data collection, rural and/or urban contexts that the data collection process was focused on, the sample size of LBC, the sample size of NLBC, sampling method(s), the gender ratio of LBC samples, the age range of LBC samples, type(s) of mental health challenge(s) addressed and the scale used to measure each type of examined mental health challenges.

**Fig 3:**
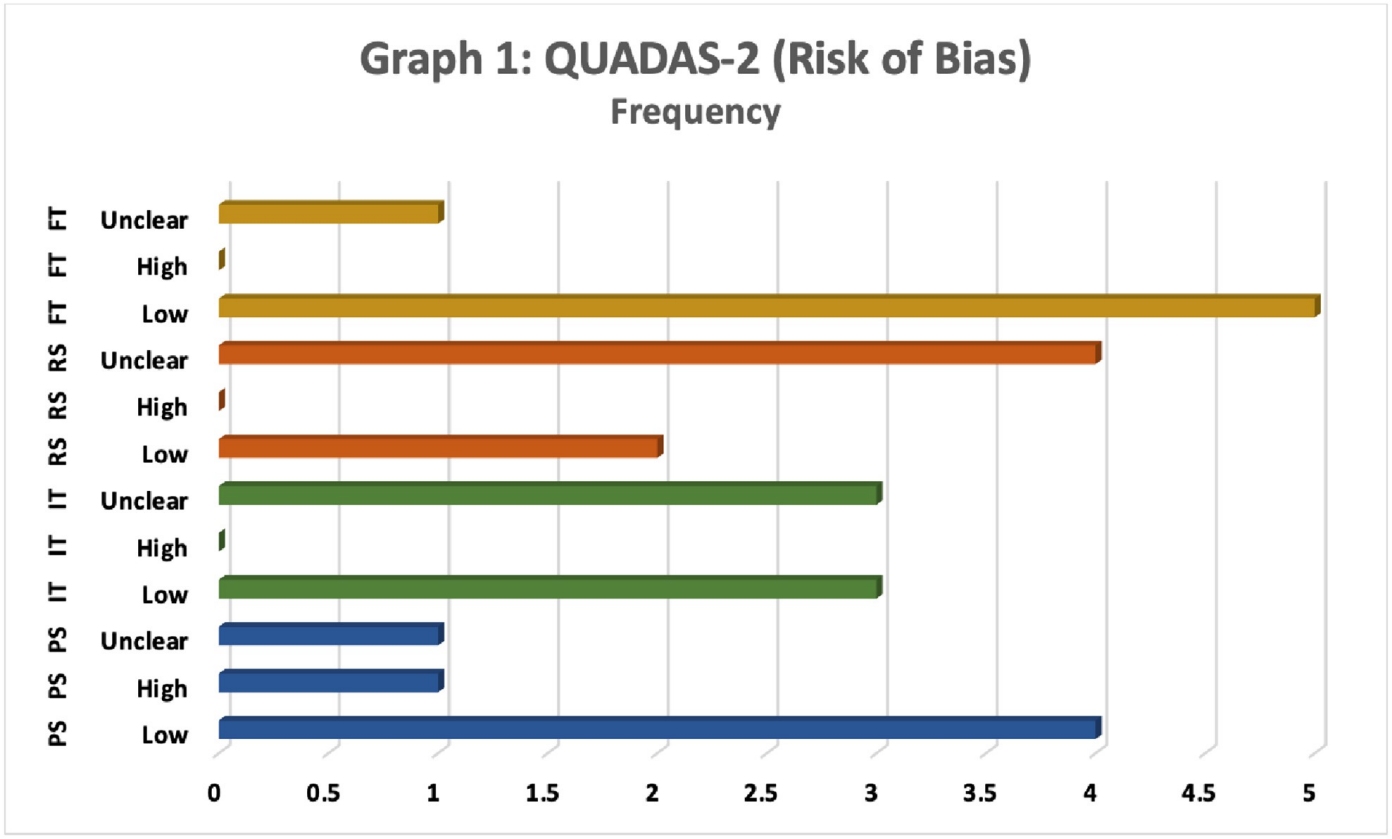
QUADAS-2 (risk of bias). Note: PS = Patient Selection; IT = Index Test(s); RS = Reference Standard; FT = Flow and Timing; “Unclear,” “High” and “Low” belong to the risk of bias

**Fig 4:**
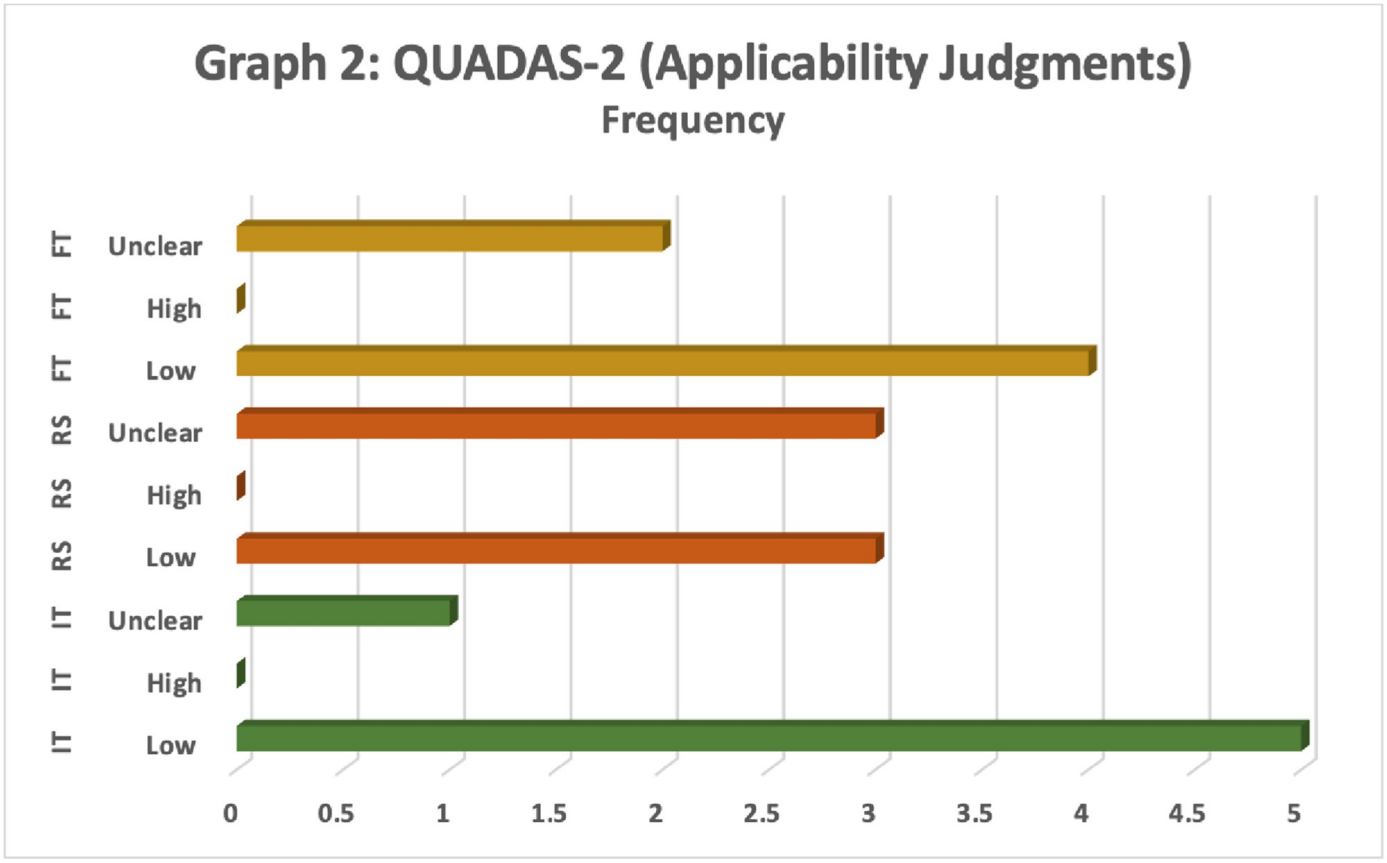
QUADAS-2 (applicability judgments). Note: IT = Index Test(s); RS = Reference Standard; FT = Flow and Timing; “Unclear,” “High” and “Low” belong to the risk of bias

The methodologic quality assessment was assessed based on the Quality Assessment of Diagnostic Accuracy Studies 2 (QUADAS– 2). Figs 3 and 4 are the bar charts that show an assessment of the quality of six studies included in the meta-analysis according to QUADAS– 2 criteria. Results for the risk of bias (see Fig 3) and concerns with respect to applicability (see Fig 4) are displayed. QUADAS– 2 criteria demonstrate that the qualities of included studies were moderate and satisfied at least four of seven domains.

## Data synthesis

### Characteristics of methodology and method

All six included studies adopted a RCT (where LBC and NLBC are treated as the experimental group and control group, respectively). All studies contain the data of the association between the demographic trait known as gender and (1) the risk of suicide, (2) depression, (3) anxiety and (4) resilience among rural Chinese LBC. Information regarding the OR and confidence intervals (CI) of all examined associations was made available. In all six studies, male samples were coded as 0 (remark: treated as the reference) and female samples were coded as 1. Each of all six studies used 95% CI (see Table 1).

**Table 1:** Summary of all included studies for meta-analysis.

No.	Reference	Data collection years	Province	R/U	LBC (n)	NLBC (n)	Sampling method	LBC: Male	LBC: Age	Mental Health Challenges	Scale
**1**	Chang, H. et al. (2017) [31]	2015	nationwide	Rural	6,034	7,918	Multi-stage cluster random	53.4%	10–18	Suicide attempts (SAs)	Information on SAs was collected by the question, ªDuring the preceding 12 months, how many times did you actually attempt suicide?º For this item, responses fall into two categories: ªneverº and ªmore than once.
**2**	Wanjie, T. et al. (2018) [15]	2017	Sichuan	Rural	1,663	1,683	Cross-sectional comparative design, random	48.0%	12–16	Depression	Kutcher Adolescent Depression Scale (KADS)
										Anxiety	Chinese version of the Screen for Child Anxiety-Related Emotional Disorders
										Psychological distress	Kessler Psychological Distress Scale (K10)
**3**	Liu, J. et al. (2016) [32]	2012	Hunan	Rural	34	34	Convenience	53.0%	4–18	Parents’ or primary caregivers’ report on children’s depression & anxiety	Child Behaviour Checklist (CBCL)
										Teachers’ report on children’s depression and anxiety	Teacher’s Report Form (TRF)
**4**	Zhao, X. et al. (2014) [33]	2011	Anhui	Rural	1,694	1,223	Random cluster	51.7%	7–17	Anxiety	Social Anxiety Scales for Children (SASC)
**5**	Liu, H. et al. (2020) [34]	2019	Shandong	Urban & Rural	312	1,293	Cross-sectional comparative design, random	39.7%	*Undergraduates (freshman—senior)	e.g. Depression, anxiety	Symptom Check-list 90 (SCL-90)
**6**	Liang, L. et al. (2018) [35]	2017	Sichuan	Rural	University students with left-behind experience (USWL) (281)	University students without left-behind experience (445)	Cluster	52.7%	*Undergraduates (freshman—senior)	e.g. Resilience	Positive Psychological Capital Questionnaire

LBC = Left-behind children; NLBC = Non-left-behind children

* In contrast to traditional data collection methods, such as questionnaires and standardized scales, in this study the researchers created a computing application to quantitively collect personal narrative texts posted by LBC on Zhihu, a Chinese question-and-answer community website. Zhihu is similar to Quora in the United States, with two functions; namely, social networking and question-and-answer (Q&A).

To perform a meta-analysis, forest plots were employed. When building forest plots, the effect size was measured by the standardised mean difference—the difference between the sample means divided by the standard deviation of the outcome [26, 27]. The authors used the I^2^ statistic to investigate the heterogeneity of effect sizes. I^2^ statistic estimates the percentage of variation among effect sizes which can be attributed to heterogeneity. A significant I^2^ statistic indicates that the degree of heterogeneity is greater than would be expected by chance [28]. A general indication of I^2^ statistics is written: within 0–40%, heterogeneity might not be crucial; within 30–60%, moderate heterogeneity might be interpreted; within 50–90%, a large degree of heterogeneity might be deemed; and within 75–100%, a tremendous level of heterogeneity might be understood [29].

To aptly conduct a rigorous meta-analysis, researchers should carry out funnel plot analyses. A funnel plot is known as a scatter plot of the effect estimates from individual studies against the measure of every study’s size or precision. In the context of this paper, the standard error of the effect estimate should be chosen as the measure of study size and plotted on the vertical axis. The OR was treated as the intervention effects, being plotted on the horizontal axis. Heterogeneity and reporting bias, among other factors, may result in an asymmetry or alternative shapes in funnel plots. If the heterogeneity satisfies the assumptions of the model, a funnel plot is arranged symmetrically and with additional horizontal scatter [30]. However, per the rule of thumb, testing funnel plot asymmetry should only be arranged given there are at least 10 studies included in the meta-analysis. This is because the power of the test is unduly low to identify the chances of real asymmetry if there are too few studies [29]. Therefore, in this paper, where only six studies were included for a meta-analysis, it is unnecessary, and the authors decided not to conduct a funnel plot.

## Results

**Fig 5:**
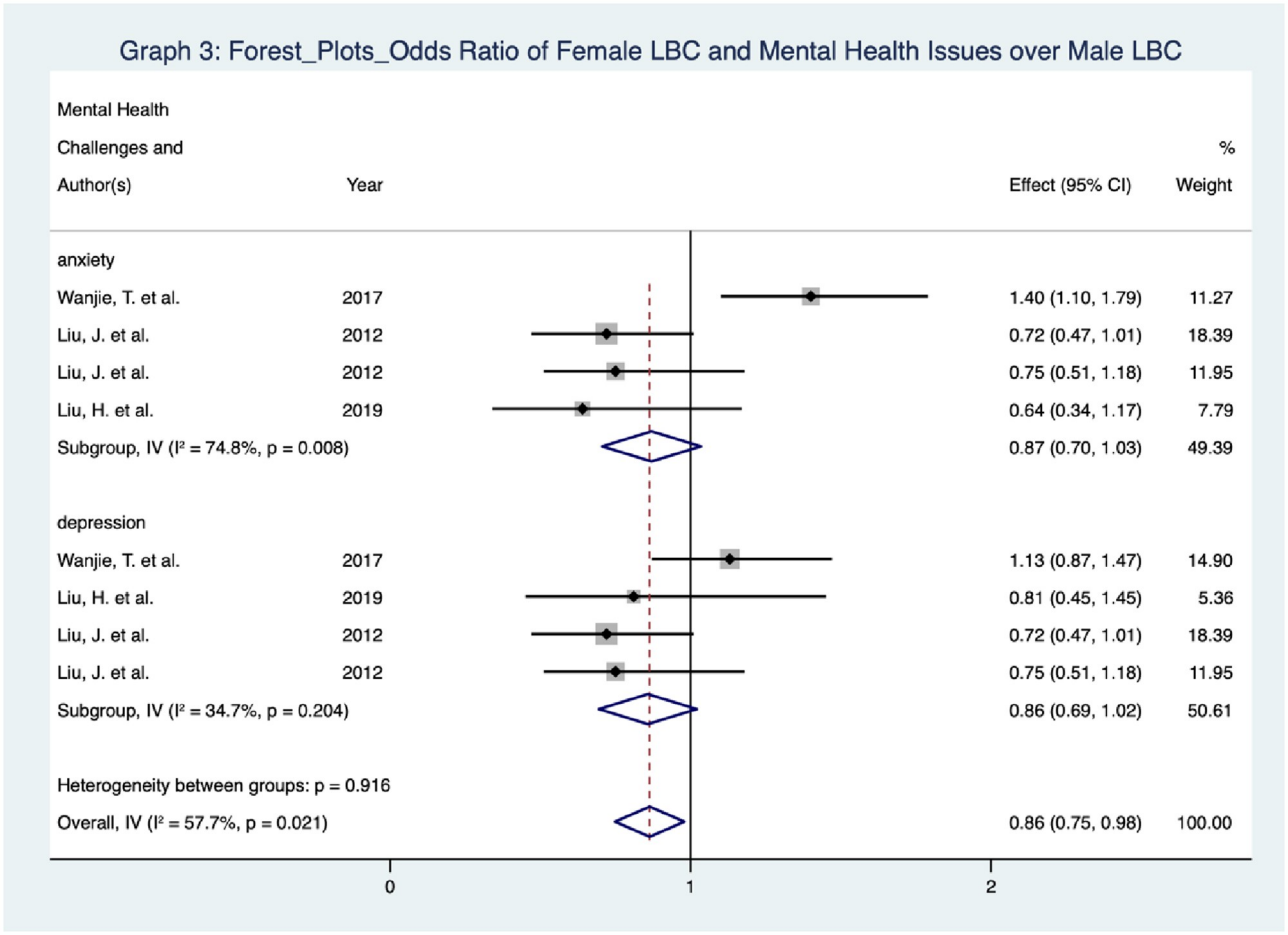
Forest plots: Odds ratio of female LBC and mental health issues over male LBC.

Heterogeneity symbolises the variation in findings that is in relation to population, risk of bias, intervention, study method and other factors of studies in meta-analyses. If significant heterogeneity is not observed, the pooled estimate is more trustworthy since most, if not all, individual studies are sharing similar findings. (1) Visual inspection of similarity of point estimates, (2) overlapping of CIs and (3) I^2^ results can be observed to test for heterogeneity [36]. From Fig 5, aside from Wanjie et al.’s [15] studies, it is noticeable that the rest of the studies share similar point estimates and their CIs overlapped to a large extent. As per the I^2^, given the presence of Wanjie et al.’s studies that demonstrate an observably higher degree of heterogeneity than the rest of the studies, the I^2^ values, each for the measurement of anxiety and depression, are 74.8 percent and 34.7 percent respectively. This shows that there is a considerable heterogeneity level for anxiety, while the heterogeneity level for depression is moderate. However, both p-values for the I^2^ statistics are larger than 0.05. Therefore, at the 0.05 significance level, it is statistically insignificant to reject the null hypothesis that there is no heterogeneity between individual studies in both the subgroups of anxiety and depression. Therefore, the concern of the potentially substantial heterogeneity should be irrelevant in this meta-analysis.

Specifically, the 95 percent CIs of all studies except one study conducted by Wanjie et al. [15] overlap one. The 95 percent CIs of the overall effect estimate, however, fail to overlap one (note: the p-value is larger than 0.05). Therefore, there is no statistical significance at the study level except for the one individual study. Apart from both studies, each measuring anxiety and depression, showing the opposite, the rest of the studies demonstrate that the intervention (i.e. being LBC) is better than the control (i.e. being NLBC). Nevertheless, again, there is no statistical significance to support such a claim at the 95 percent CI.

## Discussion

Since this meta-analysis project set up a raft of requirements when including relevant studies (such as only studies using RCTs and looking at the gendered perspective of mental health issues suffered by LBC), only a limited number of individual studies were used for data analysis. However, apart from anxiety and depression, only three studies, each for exploring psychological distress, resilience and suicide attempts, were included in the meta-analysis. Therefore, only studies addressing anxiety (5 studies in sum) and depression (4 studies in sum) were used when producing the forest plot. Statistically, there is a lack of evidence to show that the relationships between left-behind and having (1) anxiety and (2) depression are gendered.

Beyond the discussion from the forest plot, when looking at the single study addressing the relationship between being left-behind and having suicide attempts (note: LBC—OR is 1.22; 95 percent CI is 1.22 –and NLBC—OR is 1.42; 95 percent CI is 1.09–1.86 –at the p-value of 0.34), the findings demonstrate that such a relationship *per se* is not gendered at the 0.05 statistical significance level.

However, when examining the relationship between being resilient and left-behind, such an association is gendered where the OR of female left-behind university students being resilient, relative to male left-behind university students, is slightly higher than that of female non-left-behind university students being resilient, relative to their male non-left-behind university student counterparts. It is noteworthy that this study focuses on studying left-behind and non-left-behind samples who entered universities. Since a raft of LBC are socially, educationally disadvantaged, they lack the opportunities to receive higher education [37]. Therefore, the findings of this study might not be indicative of the LBC population at large.

## Conclusions

In this meta-analysis, only research projects using RCTs were included. Auhtors noticed, when conducting the systematic review, that some studies looking at the relevant gendered relationships between being left-behind and having (1) anxiety, (2) depression, (3) psychological distress and (4) suicide attempts, adopted regression analysis. In future, the authors plan to conduct another meta-analysis focusing on assessing existing studies that interpret the gendered issues grounded on regression models to see if those being left-behind, relative to the NLBC counterparts, are more psychologically vulnerable.

While the findings of this meta-analysis project fail to reflect any gendered issues statistically, the authors are aware of the fact that the data included in this project were collected based on perception. Here samples, or their parents and teachers, were responsible for answering the questions with respect to samples’ mental health status and demographic details. In China, especially in less developed rural regions, the discourse on mental health challenges might continue to be seen as taboo, so individuals giving responses might, consciously or not, tend to give socially desirable answers to avoid any potential social stigmatisation. Therefore, there is some extent of reservation regarding the validity of the included studies’ data. Unless included studies deliver data derived from objective measurement, the validity of the exploration of relationships between mental health issues and being left-behind is, to some degree, questionable.

A notable limitation of this research is the absence of searching and reviewing publications from Chinese databases, such as the CNKI Database, WANFANG Database and VIP Database. The authors developed this research as a preliminary project where only major English databases were searched. Despite such a shortcoming, the authors are currently undertaking a broader research project where both English and Chinese databases are being searched in order to address any gendered relationships between being left-behind and mental health issues in Chinese contexts. The broader, ongoing research project will expectedly be completed in 2023.

In the broader, ongoing research project, the authors, in response to the shortcomings of this systematic review and meta-analysis, are expanding the eligibility criteria when including studies for review. Here the authors not only include studies that applied RCT for data analysis, but those employed logistic regression analysis too. The authors believe by broadening the eligibility criteria, more relevant literature can be found and aptly reviewed, where such a circumstance is conducive to the performance of meta-analysis—because more studies exploring psychological distress, resilience and suicide attempts should be included.

## Supporting information

S1 Dataset The performance of the meta-analysis. (DTA)

S1 Checklist Author formatting checklist. (DOCX)
